# Destruction of Dopaminergic Neurons in the Midbrain by 6-Hydroxydopamine Decreases Hippocampal Cell Proliferation in Rats: Reversal by Fluoxetine

**DOI:** 10.1371/journal.pone.0009260

**Published:** 2010-02-17

**Authors:** Katsuaki Suzuki, Kyoko Okada, Tomoyasu Wakuda, Chie Shinmura, Yosuke Kameno, Keiko Iwata, Taro Takahashi, Shiro Suda, Hideo Matsuzaki, Yasuhide Iwata, Kenji Hashimoto, Norio Mori

**Affiliations:** 1 Osaka-Hamamatsu Joint Research Center for Child Mental Development, Hamamatsu University School of Medicine, Hamamatsu, Japan; 2 Department of Psychiatry and Neurology, Hamamatsu University School of Medicine, Hamamatsu, Japan; 3 Osaka-Hamamatsu Joint Research Center for Child Mental Development, Osaka University Graduate School of Medicine, Suita, Japan; 4 Division of Clinical Neuroscience, Chiba University Center for Forensic Mental Health, Chiba, Japan; National Institutes of Health, United States of America

## Abstract

**Background:**

Non-motor symptoms (e.g., depression, anxiety, and cognitive deficits) in patients with Parkinson disease (PD) precede the onset of the motor symptoms. Although these symptoms do not respond to pharmacological dopamine replacement therapy, their precise pathological mechanisms are currently unclear. The present study was undertaken to examine whether the unilateral 6-hydroxydopamine (6-OHDA) lesion to the substantia nigra pars compacta (SNc), which represents a model of long-term dopaminergic neurotoxicity, could affect cell proliferation in the adult rat brain. Furthermore, we examined the effects of the selective serotonin reuptake inhibitor (SSRI) fluoxetine and the selective noradrenaline reuptake inhibitor maprotiline on the reduction in cell proliferation in the subgranular zone (SGZ) by the unilateral 6-OHDA lesion.

**Methodology/Principal Findings:**

A single unilateral injection of 6-OHDA into the rat SNc resulted in an almost complete loss of tyrosine hydroxylase (TH) immunoreactivity in the striatum and SNc, as well as in reductions of TH-positive cells and fibers in the ventral tegmental area (VTA). On the other hand, an injection of vehicle alone showed no overt change in TH immunoreactivity. A unilateral 6-OHDA lesion to SNc significantly decreased cell proliferation in the SGZ ipsilateral to the 6-OHDA lesion, but not in the contralateral SGZ or the subventricular zone (SVZ), of rats. Furthermore, subchronic (14 days) administration of fluoxetine (5 mg/kg/day), but not maprotiline significantly attenuated the reduction in cell proliferation in the SGZ by unilateral 6-OHDA lesion.

**Conclusions/Significance:**

The present study suggests that cell proliferation in the SGZ of the dentate gyrus might be, in part, under dopaminergic control by SNc and VTA, and that subchronic administration of fluoxetine reversed the reduction in cell proliferation in the SGZ by 6-OHDA. Therefore, SSRIs such as fluoxetine might be potential therapeutic drugs for non-motor symptoms as well as motor symptoms in patients with PD, which might be associated with the reduction in cell proliferation in the SGZ.

## Introduction

Parkinson's disease (PD) is a chronic and progressive neurodegenerative disease with multiple motor and non-motor features that contribute to the impairment of health-related quality of life (QOL). The pathologic hallmark of PD is degeneration of dopaminergic neurons in the substantia nigra pars compacta (SNc), resulting in depletion of striatal dopamine, which regulates excitatory and inhibitory outflow of the basal ganglia [1]–[3]. With improvements in the treatment of motor symptoms, PD's non-motor symptoms (e.g., depression, anxiety, cognitive deficits, and olfactory dysfunction) have been increasingly recognized as a major cause of disability, particularly neuropsychiatric features (e.g., depression and anxiety) and cognitive impairments [4]–[11]. Depression occurs in approximately 45% of patients with PD and does not correlate with the stage of motor deficits; moreover, it reduces QOL independently of motor symptoms and it appears to be underrated and undertreated [11], [12]. In addition, non-motor symptoms, including depression and anxiety, occur not only after the onset of motor symptoms but also may develop many years, even decades, before the onset of PD, suggesting these neuropsychiatric symptoms are risk factors for the development of PD [11]–[13]. Collectively, these data suggest the need for earlier evaluation and treatment of non-motor symptoms (e.g., depression, anxiety, and cognitive deficits) in PD, which potentially could improve health-related QOL and patient productivity while reducing morbidity and minimizing direct and indirect healthcare costs [11].

Several lines of evidence suggest that serotonergic, noradrenergic, and dopaminergic mechanisms play key roles in the etiology of non-motor symptoms such as depression in PD. Antidepressants such as selective serotonin reuptake inhibitors (SSRIs) appear to be effective in treating depression in PD [11], [14]–[18]. The anti-cholinergic effects of tricyclic antidepressants are especially problematic in patients with PD, since they may also worsen cognition or aggravate orthostatic hypotension. Thus, it appears that SSRIs are generally well tolerated in patients with PD [11], [16].

Accumulating evidence suggests that the induction of neurogenesis in the hippocampus may be involved in the mechanisms of action of antidepressants such as SSRIs as well as in cognitive functions such as learning and memory [19]–[25]. Additionally, dopamine is also shown to play a role in the regulation of neural progenitor cells, because the depletion of dopamine in animal models of PD decreased the numbers of neural progenitor cells in the neurogenic regions of the brain [26]–[30]. Interestingly, Höglinger et al. [28] reported that the numbers of proliferating cells in the subependymal zone and neural precursor cells in the subgranular zone (SGZ) and olfactory bulb are reduced in the postmortem brains of patients with PD, suggesting that the generation of neural precursor cells is impaired in PD as a consequence of dopaminergic denervation. Taken together, the results suggest it is likely that reduced neurogenesis might be involved in the non-motor symptoms as well as motor symptoms in patients with PD. It is therefore suggested that increasing endogenous neurogenesis could provide a novel treatment paradigm for both motor and non-motor symptoms in patients with PD.

**Figure 1:**
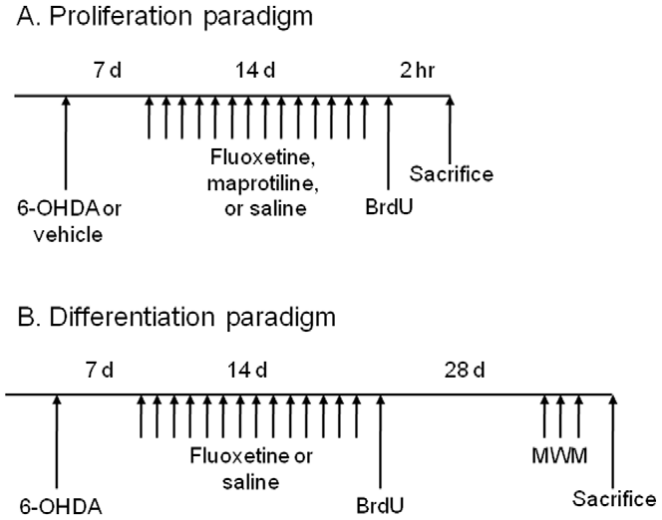
Experimental paradigms. (**A**) Proliferation paradigm. Animals received intra-SNc injection of 6-OHDA or vehicle. Seven days after the surgery, animals were injected with fluoxetine, maprotiline, or saline for 14 days. A day after the last injection, animals were given BrdU and sacrificed. (**B**) Differentiation paradigm. Animals were given intra-SNc injection of 6-OHDA. Seven days after the surgery, animals were injected with fluoxetine or saline for 14 days and given BrdU. Twenty-four days after the injection of BrdU, animals were given Morris water maze (MWM) tasks for 3 days, and then sacrificed.

The present study was undertaken to examine whether the 6-hydroxydopamine (6-OHDA)-induced unilateral lesion to the SNc, which represents a model of long-term dopaminergic neurotoxicity, could affect the number of proliferating cells in the SGZ of the dentate gyrus in the adult rat brain (**Fig. 1**). Furthermore, we examined the effects of the most widely prescribed SSRI, fluoxetine and the selective norepinephrine reuptake inhibitor maprotiline, on the reduction in the number of proliferating cells in the SGZ by the unilateral 6-OHDA lesion (**Fig. 1**).

## Results

**Figure 2:**
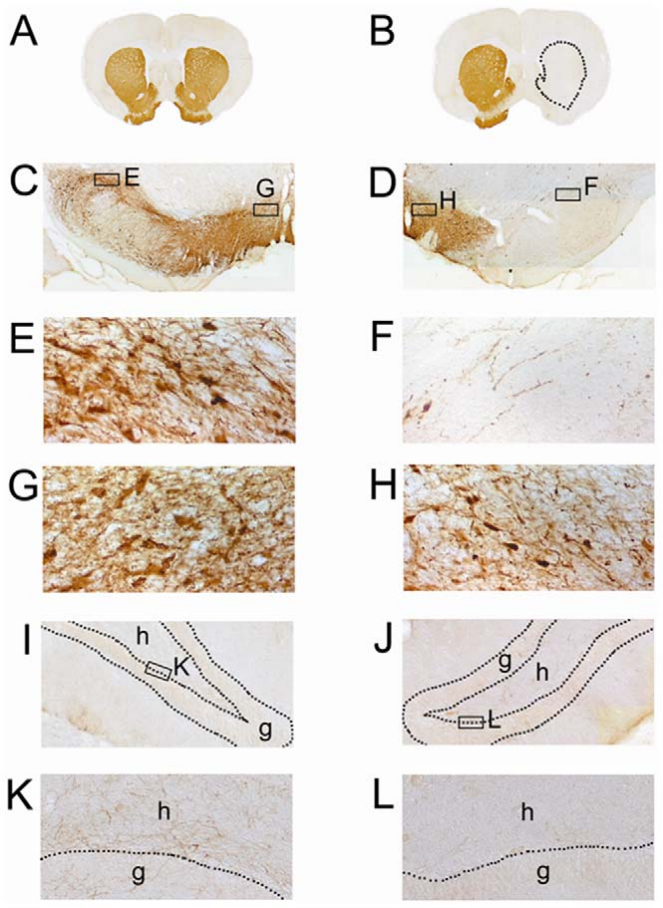
Reduced TH immunoreactivity in the striatum, midbrain, and hippocampus following 6-OHDA lesioning. (**A, B**) Coronal sections of the striatum immunostained for TH in representative animals from the sham group (A) and 6-OHDA group (B). Almost complete loss of TH immunoreactivity is observed in the right striatum (dotted area) of the 6-OHDA group animal. (**C, D**) Coronal sections of the midbrain immunostained for TH in animals from the sham group (C) and 6-OHDA group (D). (**E–H**) Sections of the SNc (E, F) and ventral tegmental area (G, H) at higher magnification of C and D. is The 6-OHDA group animal (F, H) has fewer TH-positive dopaminergic cells than the sham-operated animal (E, G). (**I–L**) Coronal sections of the hippocampus in animals from the sham group (I, K) and 6-OHDA group (J, L). TH-positive fibers are observed in the hilus (h) as well as in the granular cell layer (g) of the dentate gyrus in the sham group animal (I, K), which is completely lost in the 6-OHDA lesioned animal (J, L).

The extent of dopaminergic lesioning was determined from a series of coronal sections stained for tyrosine hydroxylase (TH) immunohistochemistry (**Fig. 2**). A single unilateral injection of 6-OHDA into the midbrain resulted in an almost complete loss of TH immunoreactivity in the striatum (**Fig. 2B**) and SNc (**Fig. 2D, F**), and in reductions in the number of TH-positive cells and fibers in the ventral tegmental area (VTA) (**Fig. 2H**), while an injection of vehicle alone showed no overt change in TH immunoreactivity (**Fig. 2A, C, E and G**). In the 6-OHDA group, TH-positive fibers in the hilus and granular cell layer of the dentate gyrus in the right hippocampus, ipsilateral to the lesioning, completely disappeared (**Fig. 2J, L**), unlike the case with that in the contralateral hippocampus (**Fig. 2I, K**).

**Figure 3:**
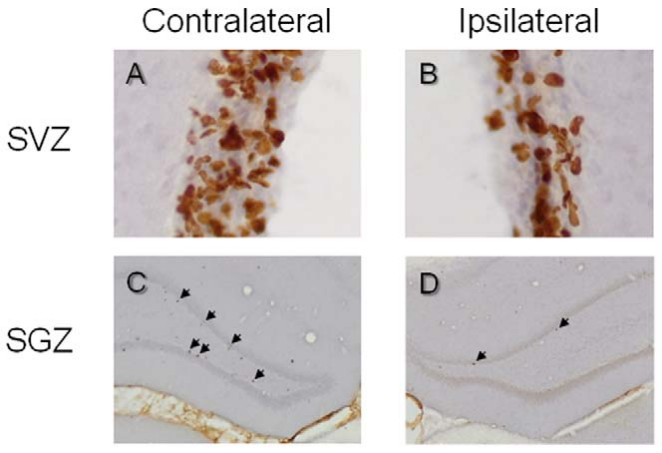
Cell proliferation in the SVZ and SGZ. (**A, B**) Coronal sections of the SVZ of the lateral ventricle contralateral (A) or ipsilateral (B) to the 6-OHDA lesioning in an animal from the 6-OHDA group. BrdU-positive nuclei are clearly observed. (**C, D**) Sections of the dentate gyrus of the hippocampus contralateral (C) or ipsilateral (D) to the 6-OHDA lesioning. BrdU-positive cells (arrows) are observed in the SGZ of the dentate gyrus.

Using BrdU immunohistochemistry, we examined the effect of the loss of dopaminergic projection on cell proliferation in the SVZ of the lateral ventricle and the SGZ of the dentate gyrus of the hippocampus. Results from a representative animal which had been lesioned by 6-OHDA and treated by subchronic saline were shown in **Fig. 3**. In the SVZ, BrdU-positive cells were distributed singly or in nested clusters throughout the SVZ (**Fig. 3A, B**). Stereological quantification revealed that the numbers of BrdU-positive cells in the SVZ were similar in both hemispheres (data not shown). In the hippocampus, BrdU-positive cells were sparsely distributed in the SGZ (**Fig. 3C, D**). 6-OHDA-induced dopaminergic denervation significantly decreased the number of BrdU-positive cells in the SGZ ipsilateral to the lesion (**Fig. 3D**).

**Figure 4:**
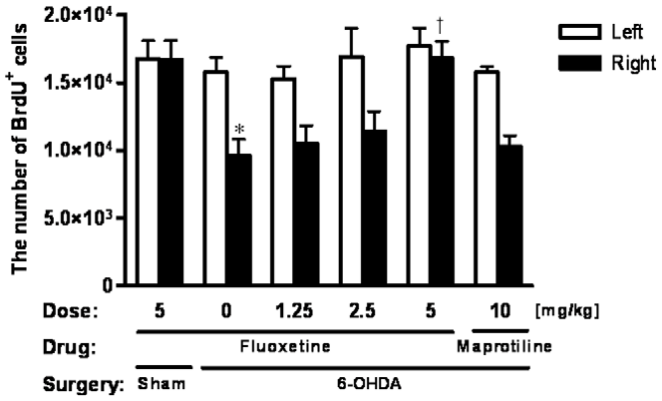
Cell proliferation in the SGZ after antidepressant treatment. Reduction of cell proliferation in animals given 6-OHDA lesioning was rescued by 2 weeks of treatment with fluoxetine (5 mg/kg) to a level comparable to that of contralateral side. *Significantly lower than the corresponding value in the contralateral side. ^†^Significantly higher than corresponding values of 6-OHDA group animals treated with saline.

We then examined the effect of subchronic treatment with fluoxetine in doses of 0 (saline), 1.25, 2.5 or 5 mg/kg/day for 14 days on the cell proliferation in the SGZ associated with a loss of dopaminergic projections. Stereological analysis in 6-OHDA-lesioned animals with saline treatment revealed fewer BrdU-positive cells in the ipsilateral SGZ than in the contralateral SGZ, and that there was no such decrease in 6-OHDA-lesioned animals treated by 5 mg/kg of fluoxetine (**Fig. 4**). Two-way ANOVA showed no significant interaction between Treatment and Side (F_5,78_ = 2.16, p = 0.07). One-way ANOVA revealed that the numbers of BrdU-positive cells differed significantly among groups on the ipsilateral side (F_5,44_ = 6.85, p<0.001) but not on the contralateral side (F_5,44_ = 0.45, p = 0.81). A *post hoc* Bonferroni test revealed significantly fewer BrdU-positive cells in the SGZ ipsilateral to the 6-OHDA lesioning in 6-OHDA group animals that received saline or 1.25 mg/kg of fluoxetine treatment than that in other conditions (p<0.01 each). BrdU-positive cells in animals treated with 10 mg/kg of maprotiline, a selective noradrenaline reuptake inhibitor, were also reduced in the ipsilateral to the 6-OHDA lesioning. This is the first report of a beneficial effect of an SSRI on the reduction in cell proliferation induced by dopaminergic lesioning of the midbrain in the adult rodent hippocampus.

**Figure 5:**
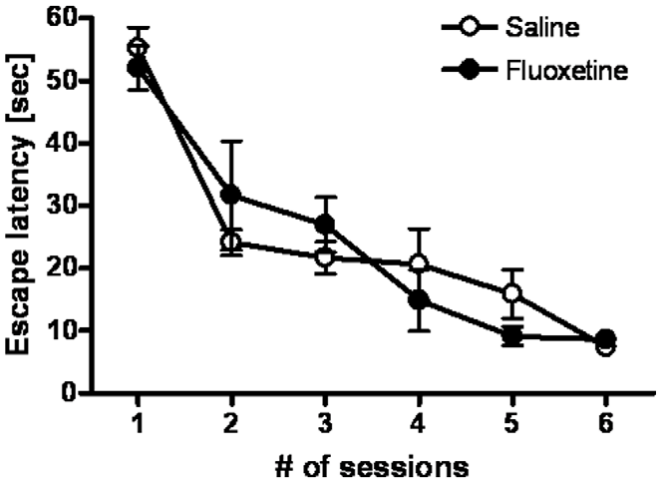
Mean escape latency in the Morris water maze task. There was no significant difference between the treatments.

We further examined the effect of fluoxetine treatment on the spatial learning task in animals with 6-OHDA lesioning. In the Morris water maze task, the escape latency to the platform in animals, which had been treated wither with saline or with fluoxetine, decreased significantly as the training progressed (repeated measure one-way ANOVA with Dunnett's multiple comparison, F_5,20_ = 11.5, p<0.01 for fluoxetine; F_5,20_ = 27.1, p<0.01 for saline), suggesting that the animals could learn the task irrespective of the drug treatment (**Fig. 5**).

**Figure 6:**
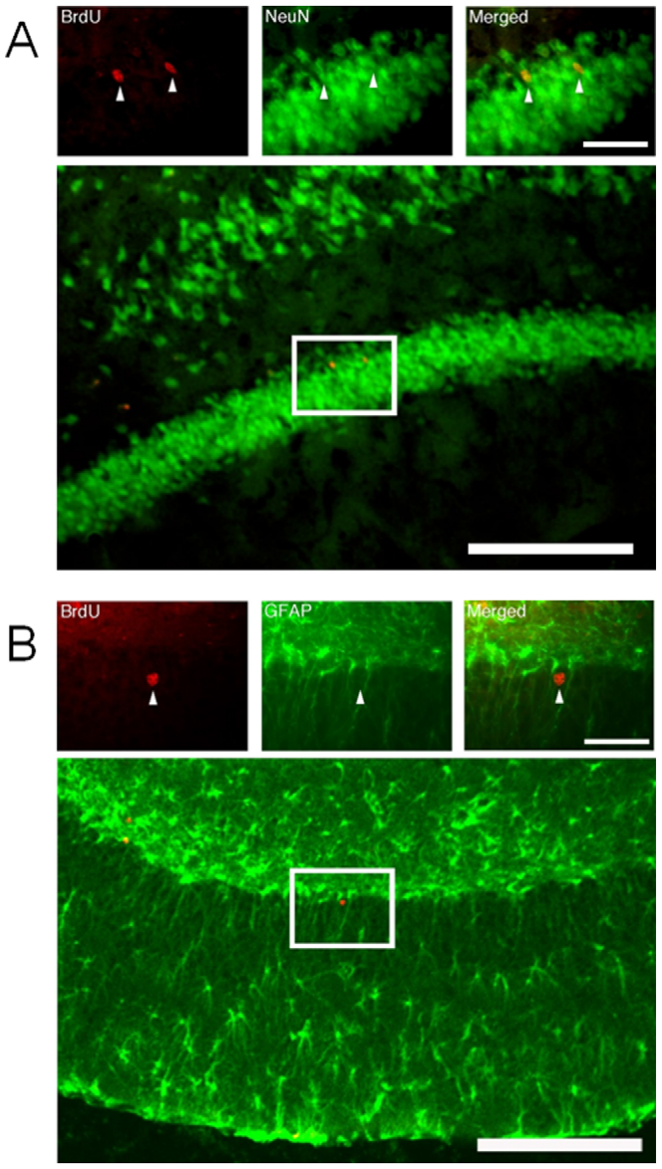
Differentiation of BrdU-positive cells in the dentate gyrus in rats 28 days after the subchronic treatment with fluoxetine. BrdU-positive cells (green) were co-labeled with a neuronal marker, NeuN (A), but not GFAP (B). Scale bars: 400 µm in lower magnification and 40 µm in higher magnification.

To detect the differentiation of surviving BrdU positive cells in animals with 6-OHDA lesioning, we used NeuN as a mature neuronal marker and GFAP as a marker for astrocytes, as shown in **Fig. 6**. Four weeks of fluoxetine treatment after the BrdU injection, the number of BrdU-positive cells in the dentate granular cell layer was 30.5±3.1 (mean ± S.E.M.) cells/mm^2^ and 18.4±3.2 cells/mm^2^ in rats treated either with subchronic fluoxetine or with saline, respectively. The difference was statistically significant as assessed by unpaired *t* test (t_8_ = 2.71, p = .027). We observed that most of BrdU-positive cells were immunoreactive to NeuN (mean ± S.E.M., 83.1±3.5%, **Fig. 6A**), whereas few BrdU-positive cells were immunoreactive to GFAP (13.9±2.5%, **Fig. 6B**) in fluoxetine treated animals.

## Discussion

The major findings of the present study are that a unilateral 6-OHDA lesion to the SNc in rat significantly decreased cell proliferation in the SGZ ipsilateral to the lesion, but not in the contralateral SGZ or SVZ, and that subchronic (14 days) administration of fluoxetine, but not maprotiline, significantly attenuated the reduction in cell proliferation in the SGZ by unilateral 6-OHDA lesion. This is a first report to demonstrate that a unilateral 6-OHDA lesion to SNc caused a reduction of cell proliferation in the SGZ ipsilateral, but not contralateral, to the lesion. These findings suggest that cell proliferation in the SGZ might be, in part, under dopaminergic control from SNc and VTA since the hippocampus receives dopaminergic input from both [31]–[33].

Accumulating evidence suggests that dopamine is a potent stimulator of endogenous neural precursor cell proliferation in the SVZ and SGZ in the adult brain [26]–[30], [34]. Höglinger et al. [28] reported the effect of dopaminergic denervation on cell proliferation in the SVZ and SGZ in the neurotoxin 1-methyl-4-phenyl-1,2,3,6-tetrahydropyridine (MPTP)-treated mice, an animal model of PD. MPTP (four injections of 10 mg/kg at 2-hour intervals) induced a complete loss of dopaminergic fibers in the SNc that fully recovered within 70 days. Cell proliferation in the SGZ first decreased and then recovered in parallel with dopaminergic denervation and reinnervation, respectively [28]. There is also a close anatomical relationship between afferent dopaminergic fibers and proliferating cells to be preserved in the SVZ of non-human primates [27]. Interestingly, the reduction in the number of proliferating precursor cells in the SVZ and SGZ of postmortem brain tissue from patients with PD was reported [28]. Furthermore, Hiramoto et al. [35] reported that dopamine receptor agonist apomorphine increased the number of BrdU-positive cells in SGZ, and that dopamine D_2_ receptor antagonist blocked the increase in BrdU-positive cells by apomorphine, suggesting that the stimulation of dopamine D_2_ receptors can increase cell proliferation in the SGZ. Taken together, the results suggest it is likely that cell proliferation in the SGZ of the dentate gyrus might be under dopaminergic control.

Freundlieb et al. [27] reported the existence of a topographically organized dopaminergic projection from the SNc to the SVZ in aged primates. MPTP treatment (four to six MPTP injections per animal; cumulative dose, 1.2–2.0 mg/kg) caused a significant decrease in the number of proliferating cells in the denervated regions of the SVZ, suggesting that an intact dopaminergic nigro-subventricular innervation is crucial for sustained neurogenesis in aged primates [27]. However, in the present study we found no alteration in the number of proliferating cells in the SVZ of adult rat brain after ipsilateral 6-OHDA lesioning. The precise reasons underlying the difference between the two studies are currently unknown. One possibility may be the differences in the degenerative profile (systemic MPTP injection (complete lesion model) vs. ipsilateral 6-OHDA lesion (partial lesion model)). Another possibility is that the intact dopaminergic neurons on the contralateral side may compensate for dopaminergic transmission to SVZ, although further study is necessary.

As mentioned, we observed that subchronic (14 days) administration of fluoxetine (5 mg/kg/day) significantly attenuated the reduction in cell proliferation in the SGZ ipsilateral to the 6-OHDA lesion, but not in the contralateral SGZ or in control rats. To the best of our knowledge, this is the first report of a beneficial effect of an SSRI on the reduction in cell proliferation induced by dopaminergic lesioning of the midbrain in the adult rodent hippocampus. Malberg and Duman [36] reported that cell proliferation in the adult hippocampus is decreased by inescapable stress, and that fluoxetine treatment (10 mg/kg b.i.d. for 7 days) blocked the downregulation of cell proliferation resulting from inescapable stress. Furthermore, repeated treatment with fluoxetine (10 mg/kg/day for 10 days) could reverse the reduction in the number of proliferating cells in the SGZ of streptozotocin (STZ)-induced diabetic mice, but not control mice [37]. Thus, it should be noted that fluoxetine can reverse the reduction in cell proliferation in the SGZ by 6-OHDA lesion, stress, or STZ. In contrast, some other reports showed that chronic administration of fluoxetine increased the number of cell proliferation in the SGZ of control rats [38]–[42], which is inconsistent with the present results. It seems that treatment schedule (e.g., dose, treatment interval) may contribute to this discrepancy, although the precise reasons underlying the discrepancy are currently unclear.

In this study, we found that, in Morris water maze task, 6-OHDA lesioning did not develop non-motor symptoms (e.g., learning and memory), suggesting that unilateral reduction in the hippocampal neurogenesis by the hemispheric dopaminergic deprivation may not be sufficient to develop behavioral non-motor symptoms (e.g., cognitive deficits). The aforementioned findings suggest that the reduction of cell proliferation in the SGZ of the adult hippocampus may be involved in the non-motor symptoms (e.g., depression, anxiety, and cognitive deficits) as well as in the motor symptoms in patients with PD. Considering the beneficial effects of SSRIs (e.g., fluoxetine) in the regulation of cell proliferation in the SGZ, it seems that SSRIs such as fluoxetine would be potential therapeutic drugs for PD. Some papers have demonstrated that antidepressants such as SSRIs are effective in the treatment of depression in patients with PD [14]–[18]. Non-motor symptoms may develop many years, even decades, before the onset of PD [11]–[13], suggesting these neuropsychiatric symptoms are risk factors for the development of PD. Taken all together, the past and present results indicate that SSRIs such as fluoxetine may delay or prevent the onset of PD in patients with prodromal non-motor symptoms. A randomized, double-blind, placebo-controlled study of SSRIs in the prodromal non-motor symptoms of PD is needed to confirm our hypothesis.

It is known that noradrenergic innervation that originates in the locus coeruleus terminates diffusely throughout the hippocampus, and one of subregions where the highest density occurs is the just inferior to the granular cell layer of dentate gyrus. Since anti-TH immunohistochemistry recognizes noradrenergic fibers, the reduction in TH immunoreactivity in the hilus of 6-OHDA treated rats (**Fig. 2L**) indicates that noradrenergic as well as dopaminergic inputs to the hippocampus have been affected by 6-OHDA treatment in our study. Axons of the locus coeruleus project to the hippocampus via the dorsal bundle, which ascends to traverse the midbrain tegmentum [43]. Due to our coordinate for the stereotaxic injection, which had intended to avoid the hippocampal injury, 6-OHDA solution along the needle track might have affected noradrenergic axons in the vicinity of the injection site. Therefore, the finding suggests that decrease in hippocampal neurogenesis might be a result from not only dopaminergic destruction but also reduction of noradrenergic inputs to the hippocampus by intra-SNc 6-OHDA. From the clinical viewpoint, the locus coeruleus appears to be constantly affected in PD, as witnessed by the extensive cell loss occurring PD patients [44]–[46]. Noradrenaline levels in several brain regions of PD patients have been shown to be significantly reduced [47]. Therefore, the ameliorating effect of SSRI on the decrease in hippocampal neurogenesis after 6-OHDA treatment implies that SSRI would be beneficial for PD patients who have deficiency in both dopaminergic and noradrenergic systems.

In conclusion, the present study suggests that cell proliferation in the SGZ of the dentate gyrus of the adult rat brain might be under dopaminergic control from SNc and VTA. Additionally, SSRIs such as fluoxetine potentially are therapeutic drugs for non-motor symptoms as well as motor symptoms in patients with PD, since fluoxetine can reverse the downregulation of cell proliferation in the SGZ by 6-OHDA lesion.

## Materials and Methods

### Animals

Adult Sprague-Dawley rats weighing 240–260 g (Japan SLC, Hamamatsu, Japan) were used in this study. The animals were housed in groups of three in a temperature- and humidity-controlled room that was kept on a 12-h light/dark cycle. Food and water were available ad libitum throughout the study. All experiments were performed in accordance with the Guide for Animal Experimentation at the Hamamatsu University School of Medicine. All efforts were made to minimize the number of animals used and their suffering. All surgeries were performed following an intraperitoneal (IP) injection of sodium pentobarbital at 50 mg/kg of body weight.

### Surgery

After at least 7 days of habituation to the rat colony, animals were divided into two groups: 6-OHDA (n = 56) and sham (n = 8). Animals in the 6-OHDA group were injected stereotaxically with 6 µg of 6-hydroxydopamine (6-OHDA, Sigma-Aldrich, Tokyo, Japan) dissolved in 2 µl of saline containing 0.02% ascorbic acid over a 10 min period into the right substantia nigra pars compacta (SNc) at the following coordinates: AP, −4.8 mm; ML, 1.5 mm with respect to the bregma; and 7.8 mm below the dura. Sham group animals were injected with the same volume of vehicle into the right SNc.

### Antidepressant Treatment

To test the effect of fluoxetine, an established antidepressant SSRI, on cell proliferation in animals given 6-OHDA, 35 animals from the 6-OHDA group and 10 animals from the sham group were used. As of the next day to the surgery as described above, the 6-OHDA group animals were injected intraperitoneally (IP) either with saline (1 ml/kg, n = 10), fluoxetine (1.25 mg/kg, n = 5; 2.5 mg/kg, n = 5; or 5 mg/ml/kg, n = 10), or with maprotiline, a selective noradrenaline reuptake inhibitor (10 mg/kg, n = 5) once daily for 14 days. Sham group animals were given 5 mg/kg of fluoxetine once daily for 14 days (**Fig. 1A**). To test the effect of subchronic treatment with fluoxetine on cell differentiation, 10 animals from the 6-OHDA group were used. As of the next day to the surgery, the 6-OHDA group animals were treated IP either with saline (1 ml/kg, n = 5) or fluoxetine (5 mg/kg, n = 5) once daily for 14 days. Animals were then allowed to survive for 28 days (**Fig. 1B**).

### Morris Water Maze Task

To determine if the fluoxetine treatment affects hippocampus dependent memory function in animals given 6-OHDA, we used a Morris water maze (MWM) task for animals in the differentiation paradigm. The animals were given a session of the task for 3 days. Each session consisted of six trials lasting 60 s each, separated by a 60 s inter-trial interval. At the start of each trial, rats were placed at one of four start locations at the limb of a circular pool (150 cm in diameter, water temperature at 26°C) with their face toward the wall. Animals were required to escape to an invisible platform (10 cm in diameter, 1 cm below the water surface) fixed at a predetermined location. If animals could not reach the platform within 60 s, an experimenter gently led them onto the platform. Once animals got upon the platform, they were left on it for 15 s and then returned to a waiting cage.

### 5-Bromo-2′-Deoxyuridine (BrdU) Labeling and Tissue Processing

Twenty-four hours after the last injection of saline, fluoxetine or maprotiline, the animals were injected with a proliferation marker BrdU (100 mg/kg, IP). Two hour or 28 days after the BrdU administration, animals were deeply anesthetized with 100 mg/kg of pentobarbital and sacrificed by transcardial perfusion with physiological saline followed by 0.1 M phosphate-buffered 4% paraformaldehyde. The brains were removed, cryoprotected by 20% sucrose in 0.1 M phosphate buffer overnight, frozen with dry ice, and cut into 30-µm-thick serial coronal sections by means of a cryostat. The sections were divided into six section series (i.e., the first series includes the 1st, 7th, 13th …, the second series includes the 2nd, 8th, 14th…, and so on) and stored in 0.1 M phosphate-buffered saline at 4°C until their use in histological examinations.

### Histology

The first series sections through the hippocampus and midbrain were stained with cresyl violet for Nissl staining to evaluate injection sites. The second series sections were immunostained for tyrosine hydroxylase (TH) in order to label soma and fibers of dopaminergic neurons. Sections were serially incubated with anti-TH polyclonal antibody (1∶300; Sigma-Aldrich Japan Inc., Tokyo, Japan) and biotinylated horse-anti-rabbit IgG (1∶500; Vector Laboratories, Burlingame, CA, USA). The third series sections were stained with mouse monoclonal anti-BrdU antibody (0.6 mg/mL; Becton Dickinson Immunocytometry Systems, San Jose, CA, USA) and biotinylated horse-anti-mouse IgG (1∶160; Vector Laboratories) to visualize proliferating cells. Immunoreactive signals were visualized using an ABC kit (Vector Laboratories) and 3,3′-diaminobenzidine (Sigma-Aldrich Japan, Tokyo, Japan).

### Immunofluorescence

The phenotype of newborn cells was examined with double-labeled immunofluorescence. The sections were denatured, washed, and incubated with the rat monoclonal anti-BrdU antibody (0.6 mg/mL; Becton Dickinson Immunocytometry Systems, San Jose, CA, USA) and subsequently with either a mouse monoclonal anti-neuronal nuclei (NeuN) antibody (1∶1000, Chemicon, Temecula, CA, USA) or a rabbit polyclonal anti-glial fibrillary acidic protein (GFAP) antibody (1∶2000, DAKO, Copenhagen-Glostrup, Denmark) at 4°C overnight. Then sections were incubated with Alexa 488 donkey anti-rat IgG (1∶1000, Molecular Probes) and with Alexa Fluor 546 goat anti-mouse IgG (1∶1000, Molecular Probes) or Alexa Fluor 546 goat anti-rabbit IgG (1∶2000, Molecular Probes) for 1 h at room temperature. Fluorescent signals were detected using a fluorescent microscope (BioZero8000, Keyence Corp., Japan).

### Cell Counts

The procedure for nonbiased stereological cell counts has been described elsewhere [48]. Briefly, BrdU-positive proliferating cells were counted on every 6^th^ section throughout the rostrocaudal extent of the brain using the StereoInvestigator system (Microbrightfield Japan, Chiba, Japan) and an Olympus BX40 microscope equipped with a motorized stage driven in the X, Y, and Z planes. BrdU-positive cells in the subventricular zone (SVZ) of the lateral ventricle and subgranular zone (SGZ) of the dentate gyrus of the hippocampus were carried out exhaustively through every 6^th^ section at 40× magnification in order to estimate the total number of cells in each structure. The volumes of the SVZ, SGZ, dentate granular layer, and hilus were estimated according to Cavalieri's principle.

In the differentiation paradigm, BrdU-positive cells were counted manually under a fluorescent microscope. At least 10 sections in the hippocampus ipsilateral to the 6-OHDA lesioning were studied in each animal treated either with fluoxetine (n = 5) or with saline (n = 5). The same areas and number of sections were studied for all the animals. The granular cell layer (GCL) was traced by using a camera lucida, and drawings were scanned and processed with NIH Image software to determine the area of the GCL at each level examined. The reference volume of the GCL was determined by summing the traced areas of GCL in each section and multiplying the result by the distance between sampled sections (180 µm). All BrdU-labeled cells in the selected area were counted in terms of cells/mm^2^.

### Statistical Analysis

The main dependent variable was the number of BrdU-positive cells. Two-way analysis of variance (ANOVA) was conducted to test the effects of ‘Treatment’ (saline, fluoxetine, and maprotiline) and ‘Side’ [right (ipsilateral to the lesion) and left] on the dependent variable. When we found significant interaction between ‘Treatment’ and ‘Side’, we subsequently repeated one-way ANOVA for each ‘Side’ separately (i.e., right versus left) to see whether any distinct patterns of the dependent variable among treatments would emerge between the sides. According to the results of one-way ANOVA, we further carried out post hoc pair comparisons using Bonferroni's test. All values and graphs are shown as mean ± standard error of the mean (SEM). All statistical analysis was performed using statistical analysis software (SPSS version 12.0J). The level of significance was set at p<0.05.

## References

[1] Samii A, Nutt JG, Ransom BR (2004). Parkinson's disease.. Lancet.

[2] Schrag A, Achott M (2006). Epidemiological, clinical, and genetic characteristics of early-onset parkinsonism.. Lancet Neurol.

[3] Weintraub D, Comella CL, Horn S (2008). Parkinson's disease - Part 1: Pathophysiology, symptoms, burden, diagnosis, and assessment.. Am J Mnag Care.

[4] Berendse HW, Booij J, Francot CM, Bergmans PL, Hijman R (2001). Subclinical dopaminergic dysfunction in asymptomatic Parkinson's disease patients' relatives with a decreased sense of smell.. Ann Neurol.

[5] Oertel WH, Höglinger GU, Caraceni T, Girotti F, Eichhorn T (2001). Depression in Parkinson's disease. An update.. Adv Neurol.

[6] Brandstädter D, Oertel WH (2003). Depression in Parkinson's disease.. Adv Neurol.

[7] Schrag A (2006). Quality of life and depression in Parkinson's disease.. J Neurol Sci.

[8] Ishihara L, Brayne C (2006). A systematic review of depression and mental illness preceding Parkinson's disease.. Acta Neurol Scand.

[9] Chaudhuri KR, Healy DG, Schapira AH, National Institute for Clinical Excellence (2006). Non-motor symptoms of Parkinson's disease: diagnosis and management.. Lancet Neurol.

[10] Schrag A, Barone P, Brown RG, Leentjens AF, McDonald WM (2007). Depression rating scales in Parkinson's disease: critique and recommendations.. Mov Disord.

[11] Weintraub D, Comella CL, Horn S (2008). Parkinson's disease - Part III: Neuropsychiatric symptoms.. Am J Manag Care.

[12] Lemke MR (2008). Depressive symptoms in Parkinson's disease.. Eur J Neurol.

[13] Gonera EG, van't Hof M, Berger HJ, van Weel C, Horstink MW (1997). Symptoms and duration of the prodromal phase in Parkinson's disease.. Mov Disord.

[14] Reijnders JSAM, Ehrt U, Weber WEJ, Aarsland D, Leestjens AFG (2008). A systematic review of prevalence studies of depression in Parkinson's disease.. Mov Disord.

[15] Dell'Agnello G, Ceravolo R, Nuti A, Bellini G, Piccinni A (2001). SSRIs do not worsen Parkinson's disease: evidence from an open-label, prospective study.. Clin Neuropharmacol.

[16] Vajda FJ, Solinas C (2005). Current approaches to management of depression in Parkinson's disease.. J Clin Neurosci.

[17] Weintraub D, Taraborelli D, Morales KH, Duda JE, Katz IR (2006). Escitalopram for major depression in Parkinson's disease: an open-label, flexible-dosage study.. J Neuropsychiatry Clin Neurosci.

[18] Devos D, Dujardin K, Poirot I, Moreau C, Cottencin O (2008). Comparison of desipramine and citalopram treatments for depression in Parkinson's disease: a double-blind, randomized, placebo-controlled study.. Mov Disord.

[19] Duman RS, Nakagawa S, Malberg J (2001). Regulation of adult neurogenesis by antidepressant treatment.. Neuropsychopharmacology.

[20] Hashimoto K, Shimizu E, Iyo M (2004). Critical role of brain-derived neurotrophic factor in mood disorders.. Brain Res Brain Res Rev.

[21] Sapolsky RM (2004). Is impaired neurogenesis relevant to the affective symptoms of depression?. Biol Psychiatry.

[22] Dranovsky A, Hen R (2006). Hippocampal neurogenesis: regulation by stress and antidepressants.. Biol Psychiatry.

[23] Sahay A, Hen R (2007). Adult hippocampal neurogenesis in depression.. Nat Neurosci.

[24] Zhao C, Deng W, Gage FH (2008). Mechanisms and functional implications of adult neurogenesis.. Cell.

[25] Perera TD, Park S, Nemirovskaya Y (2008). Cognitive role of neurogenesis in depression and antidepressant treatment.. Neuroscientist.

[26] Baker SA, Baker KA, Hagg T (2004). Dopaminergic nigrostriatal projections regulate neural precursor proliferation in the adult mouse subventricular zone.. Eur J Neurosci.

[27] Freundlieb N, François C, Tandé D, Oertel WH, Hirsch EC (2006). Dopaminergic substantia nigra neurons project topographically organized to the subventricular zone and stimulate precursor cell proliferation in aged primates.. J Neurosci.

[28] Höglinger GU, Rizk P, Muriel MP, Duyckaerts C, Oertel WH (2004). Dopamine depletion impairs precursor cell proliferation in Parkinson disease.. Nat Neurosci.

[29] Winner B, Geyer M, Couillard-Despres S, Aigner R, Bogdahn U (2006). Striatal deafferentation increases dopaminergic neurogenesis in the adult olfactory bulb.. Exp Neurol.

[30] Borta A, Höglinger GU (2007). Dopamine and adult neurogenesis.. J Neurochem.

[31] Scatton B, Simon H, Le Moal M, Bischoff S (1980). Origin of dopaminergic innervation of the rat hippocampal formation.. Neurosci Lett.

[32] Amalric M, Koob GF (1993). Functionally selective neurochemical afferents and efferents of the mesocorticolimbic and nigrostriatal dopamine system.. Prog Brain Res.

[33] Gasbarri A, Sulli A, Packard MG (1997). The dopaminergic mesencephalic projections to the hippocampal formation in the rat.. Prog Neuropsychopharmacol Biol psychiatry.

[34] Coronas V, Bantubungi K, Fombonne J, Krantic S, Schiffmann SN (2004). Dopamine D3 receptor stimulation promotes the proliferation of cells derived from the post-natal subventricular zone.. J Neurochem.

[35] Hiramoto T, Kanda Y, Satoh Y, Takishima K, Watanabe Y (2007). Dopamine D_2_ receptor stimulation promotes the proliferation of neural progenitor cells in adult mouse hippocampus.. Neuroreport.

[36] Malberg JE, Duman RS (2003). Cell proliferation in adult hippocampus is decreased by inescapable stress: reversal by fluoxetine treatment.. Neuropsychopharmacology.

[37] Beauquis J, Roig P, Homo-Delarche F, De Nicola A, Saravia F (2006). Reduced hippocampal neurogenesis and number of hilar neurones in streptozotocin-induced diabetic mice: reversion by antidepressant treatment.. Eur J Neurosci.

[38] Malberg JE, Eisch AJ, Nestler EJ, Duman RS (2000). Chronic antidepressant treatment increases neurogenesis in adult rat hippocampus.. J Neurosci.

[39] Manev H, Uz T, Smalheiser NR, Manev R (2001). Antidepressants alter cell proliferation in the adult brain in vivo and in neural cultures in vitro.. Eur J Pharmacol.

[40] Santarelli L, Saxe M, Gross C, Surget A, Battaglia F (2003). Requirement of hippocampal neurogenesis for the behavioral effects of antidepressants.. Science.

[41] Kodama M, Fujioka T, Duman RS (2004). Chronic olanzapine or fluoxetine administration increases cell proliferation in hippocampus and prefrontal cortex of adult rat.. Biol Psychiatry.

[42] Grote HE, Bull ND, Howard ML, van Dellen A, Blakemore C (2005). Cognitive disorders and neurogenesis deficits in Huntington's disease mice are rescued by fluoxetine.. Eur J Neurosci.

[43] Loughlin SE, Fallon JH, Paxinos G (1985). Locus coeruleus.. The rat nervous system. Volume 2.

[44] Gesi M, Soldani P, Giorgi FS, Santinami A, Bonaccorsi I (2000). The role of the locus coeruleus in the development of Parkinson's disease.. Neurosci Biobehav Rev.

[45] Forno LS (1996). Neuropathology of Parkinson's Disease.. J Neuropathol Exp Neurol.

[46] Greenfield JG, Bosanquet FD (1953). The brain-stem lesions in Parkinsonism.. J Neurol Neurosurg Psychiatry.

[47] Ehringer H, Hornykiewicz O (1960). Verteilung von noradrenalin und dopamine (3-hydroxytyramin) im gehirn des menschen und ihr verhalten bei erkrankungen des extrapyramidalen systems.. Klin Wschr.

[48] Wakuda T, Matsuzaki H, Suzuki K, Iwata Y, Shinmura C (2008). Perinatal asphyxia reduces dentate granule cells and exacerbates methamphetamine-induced hyperlocomotion in adulthood.. PLoS ONE.

